# Transcatheter aortic valve implantation with pre-existing mitral valve prosthesis using cardiopulmonary bypass: A case report

**DOI:** 10.1016/j.ijscr.2023.108559

**Published:** 2023-07-20

**Authors:** Yoshiki Endo, Hitoshi Nakanowatari, Akinobu Kitagawa, Yasuhisa Fukada, Yoshihito Irie

**Affiliations:** Department of Cardiovascular Surgery, Iwaki City Medical Center, 16 Kusehara Uchigo Mimayamachi, Iwaki city, Fukushima 973-8555, Japan

**Keywords:** Aortic stenosis, Balloon valvuloplasty, Case report, Cardiopulmonary support, Transcatheter aortic valve implantation

## Abstract

**Introduction:**

Transcatheter aortic valve implantation (TAVI) is rarely performed in patients with a pre-existing mitral valve prosthesis, which was excluded from the Placement of Aortic Transcatheter Valve trial. Cardiopulmonary bypass (CPB) can provide sufficient hemodynamic stability to facilitate safe implantation; specifically, we prefer using normothermic femoro-femoral CPB. Careful attention should be paid to determine the positional relationship between the two valves in such patients.

**Presentation of case:**

We present a case of transfemoral TAVI using femoro-femoral CPB in a 90-year-old female patient with a pre-existing bioprosthetic mitral valve. Baseline echocardiography revealed severe aortic valve stenosis; hence, emergency balloon valvuloplasty was performed. Three months later, elective TAVI was performed; subsequently, left ventricular ejection fraction reached 63 % without mitral valve regurgitation or stenosis.

**Discussion:**

Despite the safety of TAVI using CPB in older patients, cannula insertion into peripheral vessels carries a high risk.

**Conclusion:**

As CPB can increase tissue invasion for a short duration, the safety of this procedure needs further validation.

## Highlights

• We report a transfemoral TAVI case with femoro-femoral CPB in a 90-year-old patient.
• Cannula insertion into peripheral vessels carries a high risk.
• CPB can increase tissue invasion for a short duration.

## 1 Introduction

Transcatheter aortic valve implantation (TAVI) is an alternative treatment strategy for severe aortic valve (AV) stenosis in patients who require surgery but for whom surgery is contraindicated due to comorbidities [1]. However, limited data exist regarding TAVI for patients with pre-existing mitral valve (MV) prostheses [1]. In these patients, TAVI may increase the risk of hemodynamic collapse. Thus, we have tried to implement cardiopulmonary bypass (CPB) for high-risk patients undergoing other cardiac procedures to prevent hemodynamic collapse. TAVI using CPB is conducted to reduce life-threatening complications and provide sufficient time for accurate AV deployment. Herein, we present a case of transfemoral TAVI using CPB.

This report was written in accordance with the SCARE criteria [2].

## 2 Presentation of case

A 90-year-old woman with chronic dyspnea, bilateral lower limb edema and weight gain was admitted to our institution. Ten years earlier, she had undergone MV replacement with the Carpentier–Edwards Perimount Bioprosthetic Valve (Edwards Lifesciences, Inc., Irvine, CA, USA) for MV stenosis due to rheumatic fever and concomitant tricuspid valve annuloplasty for regurgitation. A chest radiograph showed bilateral pleural effusion. Baseline echocardiography revealed severe AV stenosis, an AV area measuring 0.96 cm^2^, mean AV gradient of 47 mm Hg, and left ventricular ejection fraction (LVEF) of 59.7 %. We diagnosed the patient as having severe AV stenosis and considered her symptoms as being derived from it. The surgical mortality risk was 8.41 % according to EuroSCORE-II [3] and 15.8 % according to the Society of Thoracic Surgeons' score [3]; thus, our heart team recommended TAVI.

On admission, the patient presented with heart failure, which was resolved by emergency balloon valvuloplasty (BAV) with a 20 × 40-mm balloon. Preprocedural computed tomography with multi-planar reconstruction estimated the distance from the aortic annulus to the mitral metallic frame (mitro-aortic space) to be 5.4 mm. The AV annulus area was 436 mm^2^. The patient left the hospital, and TAVI was performed electively 3 months later. Echocardiography revealed an aortic valve area of 0.65 cm^2^, mean AV gradient of 50 mm Hg, and LVEF of 62 %, and the EuroSCORE-II [3] was 7.37 %. A 20 × 40-mm balloon was placed on the left ventricular outflow using CPB to confirm that it would not interfere with the MV prosthesis. Transesophageal echocardiography did not identify any malfunction. Under hemodynamic stabilization by femoro-femoral CPB, TAVI was performed with a 23-mm SAPIEN 3 valve (Edwards Lifesciences, Inc.) and was completed without any complications. Postoperatively, the patient had stable daily urinary output and lost weight steadily. Echocardiography showed that the mean AV gradient decreased to 15 mm Hg without regurgitation. The patient's LVEF reached 63 % without MV regurgitation or stenosis, and she was classified as New York Heart Association Class I [4]. The patient was discharged on postoperative day 8 and showed improvement of symptoms.

Written informed consent was obtained from the patient for the publication of her anonymized information. A copy of the written consent form is available for review by the Editor-in-Chief of this journal upon request.

## 3 Discussion

**Fig. 1:**
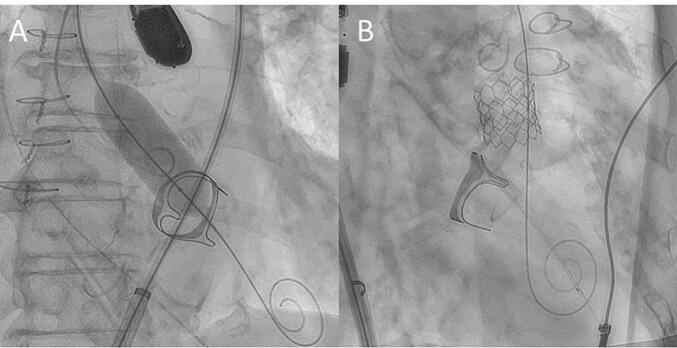
(A) Intraprocedural observation of the MV prosthesis during BAV is suggested for assessing feasibility immediately before AV bioprosthesis implantation. (B) The AV was safely implanted without interference. AV, aortic valve; BAV, balloon valvuloplasty; MV, mitral valve.

In this study, we present a case of transfemoral TAVI using CPB. The Placement of AoRTic TraNscathetER Valve (PARTNER) trial considered MV prosthesis as an exclusion criterion [1]; thus, data on TAVI with MV prostheses remain limited. As surgical techniques for TAVI improve, it is becoming possible to treat even older patients who have undergone open-heart surgery. Although TAVI is possible in experienced high-volume institutions even without CPB, we propose TAVI using CPB as a safe and reliable treatment method in any institute. CPB during TAVI provides hemodynamic stability, rendering the procedure safer while providing sufficient time for appropriate valve positioning [[4], [5], [6], [7], [8], [9]]. As our team is experienced in performing SAPIEN 3 AV prosthesis, we conducted this unique procedure. During TAVI in cases of pre-existing MV prostheses, it is important to pay attention to any interference or deformation of the prosthetic mitral leaflets, ensuring that the aortic bioprosthesis is expanded without distortion of the valve housing, device embolization from stent migration, or distortion of the mitro-aortic space (Fig. 1).

**Fig. 2:**
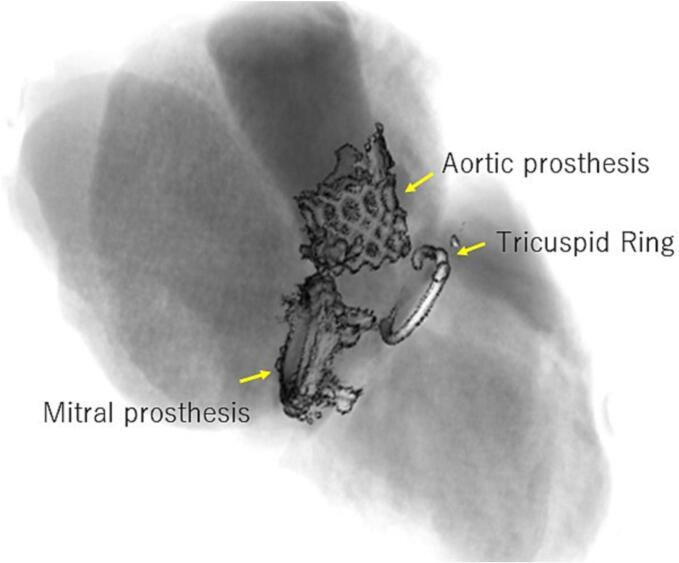
The type of the pre-existing MV is important for TAVI. A mitral bioprosthesis with longer struts protrudes into the left ventricle. MV, mitral valve; TAVI, transcatheter aortic valve implantation.

The pre-existing MV type is important because it can potentially interfere with the AV bioprosthesis. Low-profile mechanical prostheses protrude into the left ventricle less than mitral bioprostheses with longer struts (Fig. 2). Intraprocedural observation of the MV prosthesis during BAV is recommended for assessing feasibility immediately before TAVI [10].

Insertion of the femoro-femoral bypass cannula into the peripheral vessels is a high-risk procedure, especially in patients with AV stenosis, because of arterial calcifications that can cause dissection. Moreover, although TAVI can be performed safely in older patients, CPB can increase the tissue invasion for a short duration; thus, the safety of TAVI using CPB needs further verification.

## 4 Conclusion

Although TAVI with cardiopulmonary bypass in older patients may be safe, insertion of the cannula into peripheral vessels carries a high risk. Furthermore, even short-duration cardiopulmonary bypass can increase tissue invasion. TAVI is feasible in cases of pre-existing MV prostheses. However, prospective long-term follow-up data are needed to establish the risks associated with concomitant CPB.

## Informed consent

Written informed consent was obtained from the patient to publish her anonymized information.

## Ethical approval

Ethical approval to report this case was obtained from the ethics committee of Iwaki City Medical Center (No: R4-54: December 22, 2022).

## Funding

This research did not receive any specific grant from funding agencies in the public, commercial, or not-for-profit sectors.

## Guarantor

Yoshiki Endo.

## Research registration number

This is a case report and, therefore, no registration was required.

## CRediT authorship contribution statement

**Yoshiki Endo:** Conceptualization, Investigation, Writing – original draft, Writing – review & editing. **Hitoshi Nakanowatari:** Conceptualization, Investigation. **Akinobu Kitagawa:** Conceptualization, Investigation. **Yasuhisa Fukada:** Conceptualization, Investigation. **Yoshihito Irie:** Conceptualization, Investigation, Writing – review & editing.

## Declaration of competing interest

None.
